# Colonic endometriosis: from subtotal bowel obstruction to malignant transformation - a case series and literature review

**DOI:** 10.1186/s12957-025-03888-x

**Published:** 2025-06-11

**Authors:** Roland Fejes, Zsófia Balajthy, Csaba Góg, Ágota Vajda, Fanni Hegedűs, Zsolt Simonka, Szabolcs Ábrahám

**Affiliations:** 1 Institute of Surgical Research, Albert Szent-Györgyi Medical School, University of Szeged, Szőkefalvi-Nagy Béla Street 6, Szeged, H-6720 Hungary; 2 Department of Internal Medicine, Hódmezővásárhely-Makó Healthcare Center, Makó, Hungary; 3 Department of Internal Medicine, Hódmezővásárhely-Makó Healthcare Center, Hódmezővásárhely, Hungary; 4 Department of Pathology, Albert Szent-Györgyi Medical School, University of Szeged, Szeged, Hungary; 5 Department of Surgery, Albert Szent-Györgyi Health Center, University of Szeged, Szeged, Hungary

**Keywords:** Endometriosis, Endometrioid adenocarcinoma, Gastrointestinal malignancy, Colonoscopy, Lower gastrointestinal bleeding, Colorectal surgery

## Abstract

**Background:**

Colonic involvement due to endometriosis is a rare condition with a nonspecific clinical presentation. In rare instances, it may undergo malignant transformation, mimicking primary colorectal carcinoma and complicating clinical decision-making.

**Case presentation:**

We present two cases illustrating the diverse clinical manifestations of colonic endometriosis. In Case 1, a female patient underwent appendectomy for abdominal pain, but further evaluation revealed full-thickness endometriosis of the sigmoid colon, causing subtotal occlusion. In Case 2, sigmoid endometriosis was discovered during endoscopic evaluation prompted by positive occult fecal blood testing. Histopathological analysis revealed malignant transformation to endometrioid adenocarcinoma. In both cases, definitive treatment was achieved via laparoscopic sigmoid resection, highlighting the role of laparoscopic surgery in managing such conditions.

**Conclusions:**

The potential for malignant transformation of colonic endometriosis and its tendency to mimic colorectal carcinoma underscore the importance of proper tissue sampling methods and histopathological confirmation. A high index of suspicion and appropriate surgical intervention are key to effective management.

## Background

Endometriosis is a common gynecological disorder characterized by the growth of ectopic endometrioid tissue outside the uterus [1]. The aberrant tissue can implant in various anatomical locations, with the pelvis being the most frequently affected site. However, extrapelvic locations, including the gastrointestinal (GI) tract, urinary bladder, lungs, central nervous system, and skin, can also be involved, albeit rarely [2, 3]. Although uncommon, GI involvement occurs most frequently in the rectosigmoid region, accounting for 50–90% of intestinal endometriotic cases [4]. The primary clinical challenge in these cases stems from the nonspecific symptoms, including dyschezia, abdominal pain, altered bowel habits, and GI bleeding. Deeply infiltrating lesions can mimic primary colorectal malignancies, leading to severe complications such as subtotal or total bowel occlusion. The extraovarian malignant transformation of endometriosis is rare, with the first reported case dating back to 1925. Since then, about 20 cases of endometrioid adenocarcinoma (EAC) in the GI tract have been documented [5, 6]. In this work, we present two cases of deeply infiltrating colonic endometriosis. In Case 1, an initial misdiagnosis was followed by a thorough internal medicine differential diagnostic process, which eventually revealed the true underlying cause: endometriosis leading to subtotal colonic obstruction. Case 2 involved a rectosigmoid mass presumed to be primary colorectal carcinoma (CRC), but histopathological examination revealed EAC in the sigmoid colon. The primary aim of this work is to contribute to medical literature through a detailed presentation of two cases, which may aid in the recognition of a delicate condition and facilitate the rapid identification of non-colorectal, benign and malignant pathologies causing colonic obstruction.

## Case presentation

### Case 1

**Fig. 1:**
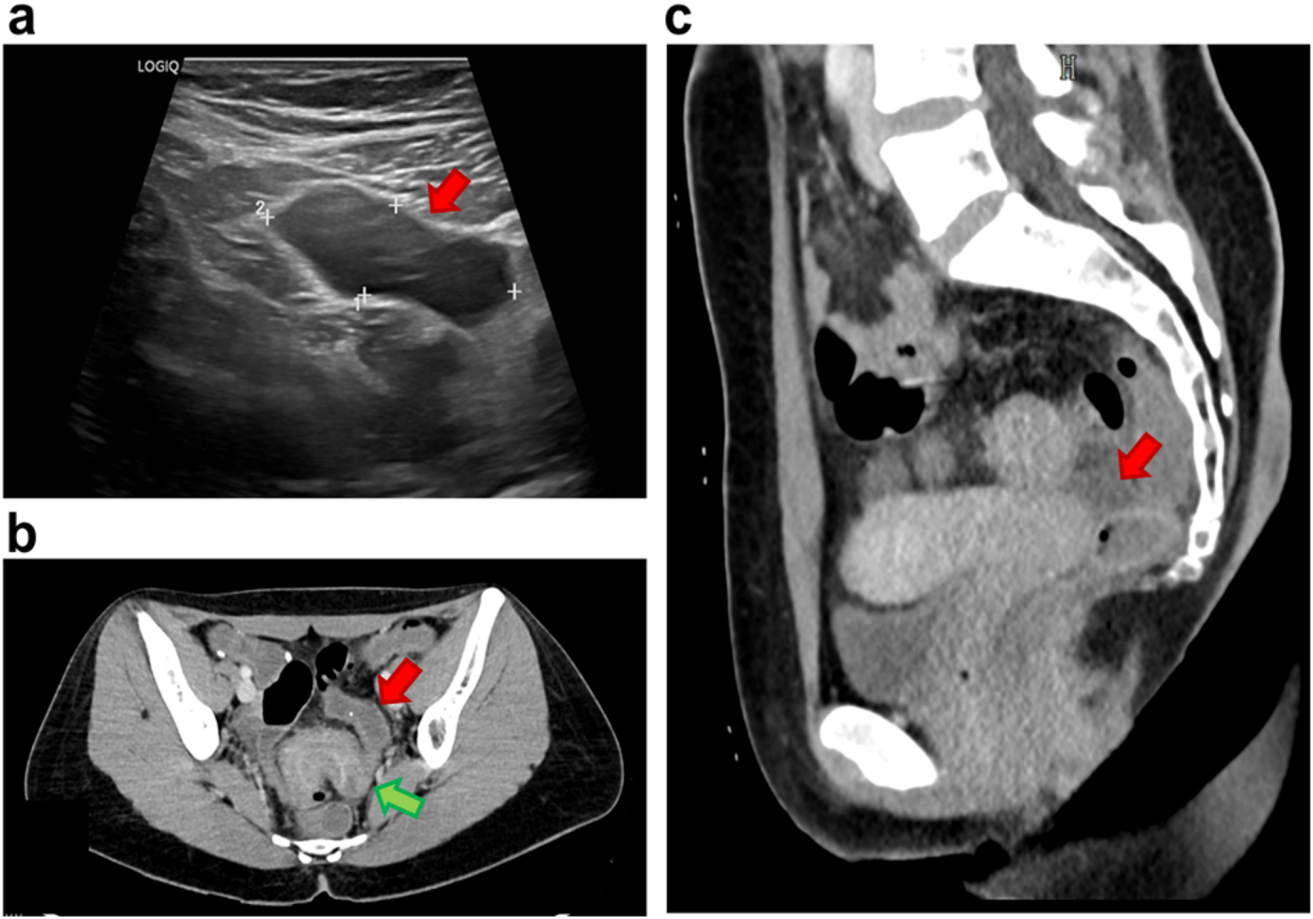
Key radiological findings in Case 1. **(a)** Ultrasound image showing a 20 × 40 mm anechoic fluid collection in the expected projection of the vermiform appendix (red arrow), which itself is not visualized (red arrow). **(b)** Contrast-enhanced axial CT scan of the pelvis: The sigmoid colon shows an S-shaped oral segment (red arrow) and a horseshoe-shaped aboral segment (approximately 8–10 cm) (green arrow). The wall of the aboral segment shows significant circumferential thickening, narrowing the lumen. However, the degree of patency cannot be assessed due to the absence of contrast material (the patient vomited before the examination). **(c)** Contrast-enhanced sagittal CT scan of the pelvis: The uterus and ovaries appear normal. Pathologically enlarged lymph nodes are visible in the examined region. A minimal amount of free abdominal fluid is seen around the sigmoid colon (red arrow)

**Fig. 2:**
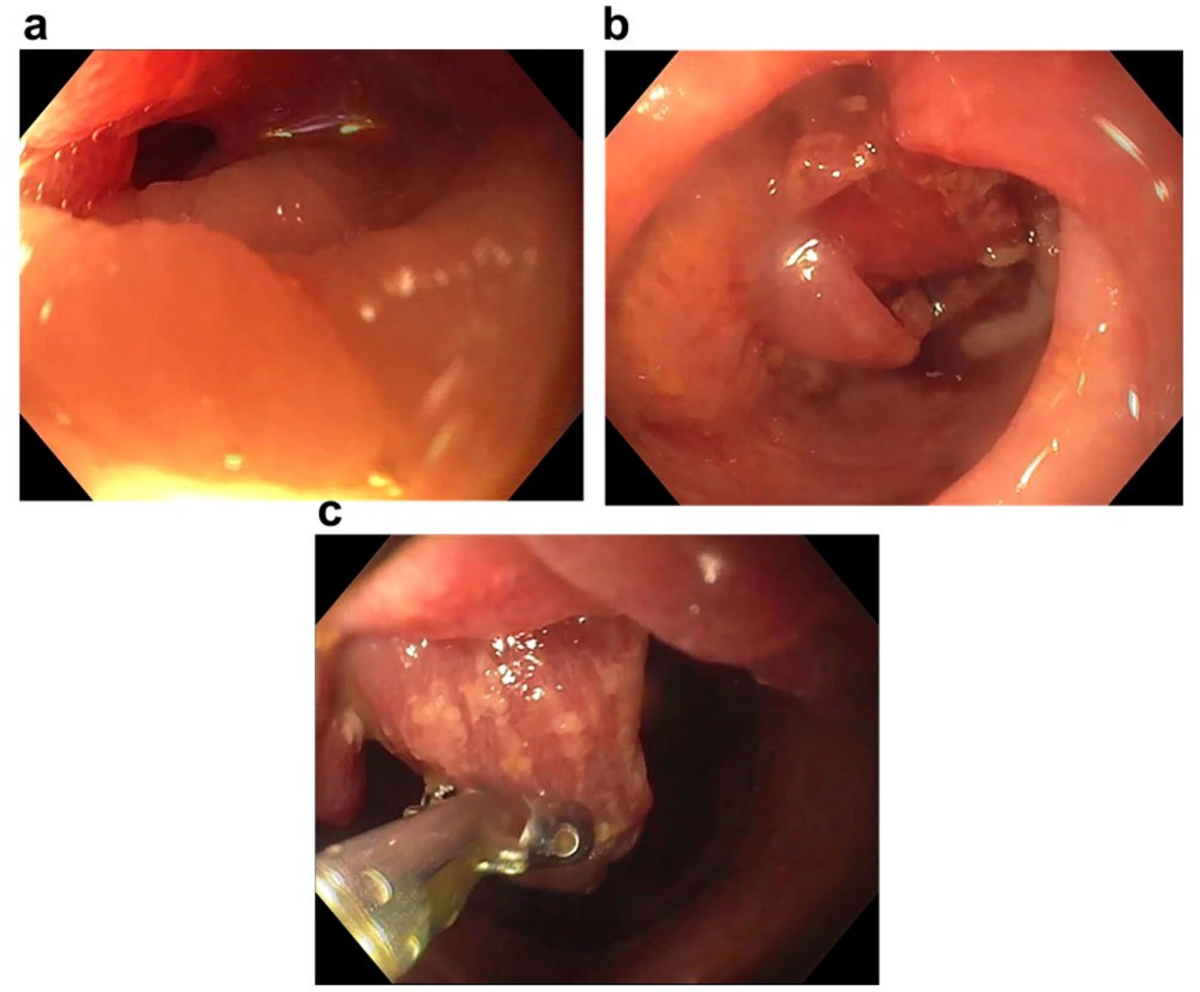
Colonoscopic findings in Case 1. The procedure was performed under intravenous anesthesia, with a 23 cm insertion length achieved, and bowel preparation scored 0-1-2 on the Boston Bowel Preparation Scale. **(a)** In the sigmoid colon, the lumen showed circumferential infiltration of the polypoid by foreign tissue, resulting in critical luminal narrowing, preventing the passage of the colonoscope. **(b)** Beyond the approximately 7–8 cm long stricture, multiple polypoid lesions with livid mucosal discoloration were observed using a gastroduodenoscope. The possibility of intussusception was also considered. Orally, the hepatic flexure was reachable; no pathological findings were observed in this segment. **(c)** Five biopsies were taken from polypoid lesions

**Fig. 3:**
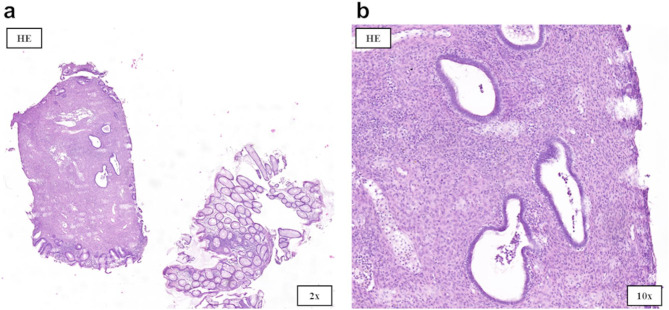
Histopathological examination of endoscopic biopsies in Case 1. **(a)** Low-power view (2×); **(b)** higher magnification (10×). In 2 of the 5 endoscopic samples, cystically dilated endometrial glands surrounded by stroma were visible alongside intact intestinal mucosa. No signs of dysplasia or malignancy are observed

Investigations: A 27-year-old woman with no chronic illnesses or regular use of medications presented to the emergency department with lower abdominal pain and nausea. Based on laboratory findings (Table 1), tenderness at the McBurney’s point, and the presence of fluid in the ileocecal region on abdominal ultrasound (Fig. 1a), a laparoscopic appendectomy was performed for suspected appendicitis; however, intraoperatively, the appendix did not appear inflamed. The patient’s symptoms persisted postoperatively, leading to further gastroenterological evaluation. Stool cultures, *Helicobacter pylori* serology, hydrogen breath tests, and food allergy panel all yielded negative results (Table 1). However, three positive fecal occult blood tests (FOBT), and elevated calprotectin levels suggested ongoing GI bleeding. These findings prompted a colonoscopy, which revealed multiple semipedunculated polyps and a circumferential infiltrative lesion causing critical stenosis in the distal third of the sigmoid colon (Fig. 2a–c). Five punch biopsies were taken from the stenotic segment. Given the suspicion of intussusception, urgent contrast-enhanced abdominal computed tomography (CT) was performed, which ruled out intussusception or malignancy (Figs. 1b and c). However, histopathological analysis of the colonoscopic biopsy samples revealed endometriosis (Figs. 3a and b).

**Table 1:** Major laboratory findings in the cases presented in this work

Parameter	Case 1	Case 2	Reference
White blood cell count (G/l)	6.3	7.2	4–10
Neutrophil (%)	67.4	55.1	42–75
Hemoglobin (g/l)	121	**109**	120–150
Hematocrit (%)	36.6	38.9	35–45
Mean corpuscular volume (fl.)	90.8	**76.4**	80–95
Thrombocyte count (G/l)	171	322	100–400
International Normalized Ratio	0.87	1.06	0.80–1.10
Blood urea nitrogen (mmol/l)	4.1	6.6	3.0–8.0
Creatinin (µmol/l)	71	77	50–100
Glomerular filtration rate (ml/min)	> 90	> 90	> 90
Total bilirubin (µmol/l)	8.7	16.1	5.0–20.0
Aspartate transaminase (U/l)	14	17	5–45
Alanine transaminase(U/l)	7	11	5–45
Gamma-glutamyl transferase (U/l)	10	11	7–32
Alpha-amylase (U/l)	26	42	30–100
Alkaline phosphatase (U/l)	79	36	35–104
Lactate dehydrogenase (U/l)	292	301	240–480
Sodium (mmol/l)	144	144	135–145
Potassium (mmol/l)	4.0	3.9	3.5–5.2
C-reactive protein (mg/l)	**23**	9.7	0.1–10.0
Glycated hemoglobin A1c (%)	5.4	5.1	4.0–6.0
Total protein (g/l)	66	78	60–80
Albumin (g/l)	44	42	35–50
Iron (µmol/l)	16.3	12.3	9–30
Ferritin (ng/ml)	28	17	10–291
Transferrin (g/l)	2.7	3.0	2.0–4.0
Transferrin saturation (%)	24.4	21.1	---
IgA (g/l)	1.23	n/a	0.9–4.5
Tissue transglutaminase (U/ml)	< 3	n/a	< 3
IgG (g/l)	9.96	n/a	8.0–17.0
Anti-*Saccharomyces cerevisiae* antibody (U/ml)	< 20	n/a	< 20
Fecal occult blood test	**3/3**	**3/3**	---
Stool calprotectin (µg/g)	**489**	n/a	80–160
Carcinoembryonic antigen (ng/ml)	n/a	**6**	< 3
Carbohydrate Antigen 19 − 9 (U/ml)	n/a	28	< 37
Cancer Antigen 125 (U/ml)	n/a	**92**	< 35

**Fig. 4:**
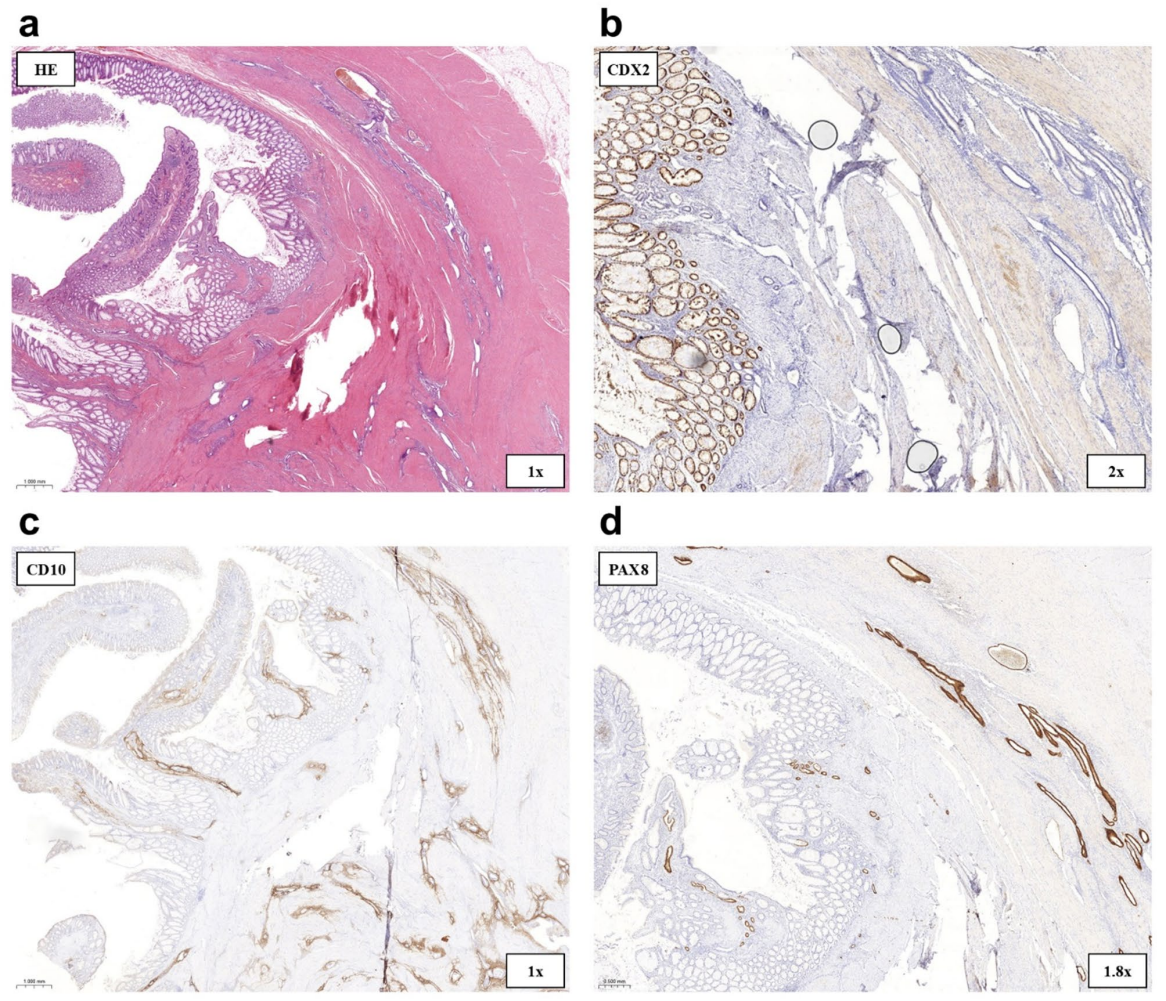
Histopathological examination of the postoperative specimen in Case 1. **(a)** Macroscopic examination shows a thickened, fibrotic intestinal wall with reddish-brown areas and a 37 × 26 mm grayish-white, livid-appearing polypoidal mucosal growth protruding into the lumen. Histopathologically, endometrial glandular metastases are observed throughout the full thickness of the intestinal wall, accompanied by endometrial stroma, with cystically dilated glands. **(b)** CDX2 immunohistochemistry shows diffuse 3 + nuclear positivity in the colorectal mucosa, while the ectopic endometrial glands are CDX2-negative. **(c)** Diffuse expression of 2 + CD10 is seen in the endometrial stroma in the intestinal wall and beneath the colorectal mucosa. **(d)** Diffuse PAX8-positive endometrial glands (Müller tube origin)

Treatment: Due to subtotal obstruction, semiurgent laparoscopic surgical management was planned. The surgical procedure involved coagulation of an endometriotic plaque on the left ovary and sigmoid resection. Gastrointestinal continuity was restored with an end-to-side anastomosis. Histopathological examination of the resected specimen confirmed endometriosis, with endometrial glands and stroma infiltrating the full thickness of the colonic wall (Fig. 4a–d). Following surgery, the patient became completely asymptomatic.

### Case 2

**Fig. 5:**
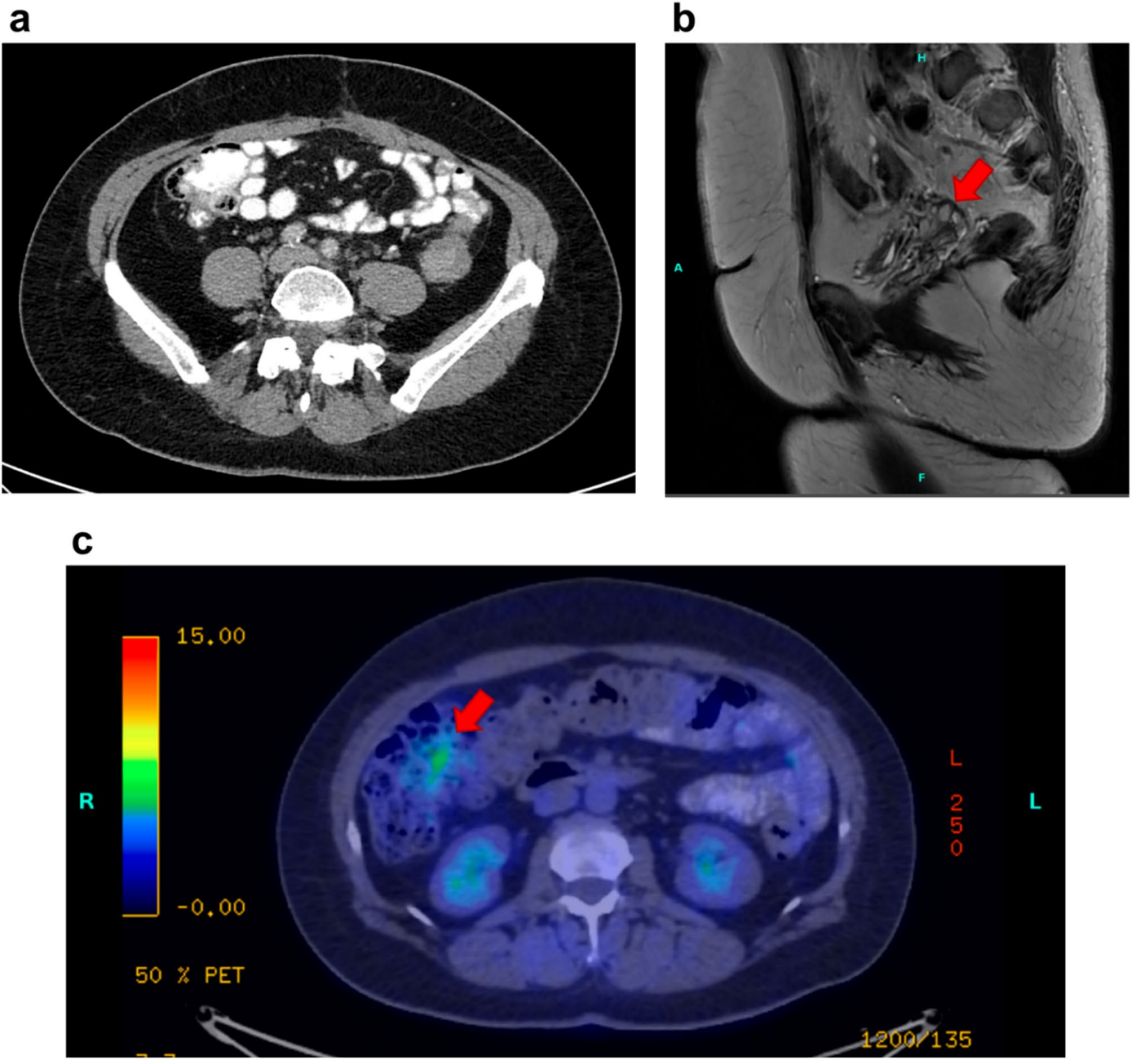
Major radiological findings in Case 2. **(a)** Contrast-enhanced axial CT scan of the pelvis showing post-hysterectomy changes with diffuse cystic formations. **(b)** Contrast-enhanced sagittal MR scan of the pelvis shows multiple cystic lesions, including an 18 mm lesion cranial to the bladder (red arrow) and a 10 mm lesion in the vicinity of the rectum. A 30 × 22 × 40 mm lobulated lesion with contrast-enhancing components is seen at the rectosigmoid junction, appearing polypoid with partial infiltration of the sigmoid colon wall. Some diverticula are also visible in the sigmoid colon. No pelvic follicle or abnormal lymph nodes are visible. **(c)** Low-dose contrast-enhanced sagittal PET/CT scan of the pelvis showing diffuse cystic lesions with FDG accumulation in the rectosigmoid area

Investigations: A 43-year-old woman presented with generalized weakness, weight loss, and abdominal pain, prompting a diagnostic workup. Her medical history included a previous surgery for right ovarian endometriosis with diffuse local infiltration and a uterine myoma, during which she underwent adhesiolysis, right adnexectomy, left salpingectomy, and a Chrobak-type partial hysterectomy, 5 years prior. Laboratory tests showed mild microcytic anemia and three consecutive positive FOBTs(Table 1). A lower GI endoscopy revealed a pedunculated polypoid lesion nearly occluding the lumen, located approximately 15 cm from the anal verge, and biopsies were taken from the lesion. Initial histopathological examination suggested infiltration by a serous carcinoma of ovarian origin. Gynecological evaluation revealed a 20-mm round cystic lesion above the vaginal vault, confirmed by contrast-enhanced abdominal CT. Further evaluation with positron emission tomography CT and pelvic magnetic resonance imaging showed a suspicious lesion in the sigmoid colon wall, likely malignant, adjacent to the previously identified, but no distant metastases were detected (Fig. 5a–c).

**Fig. 6:**
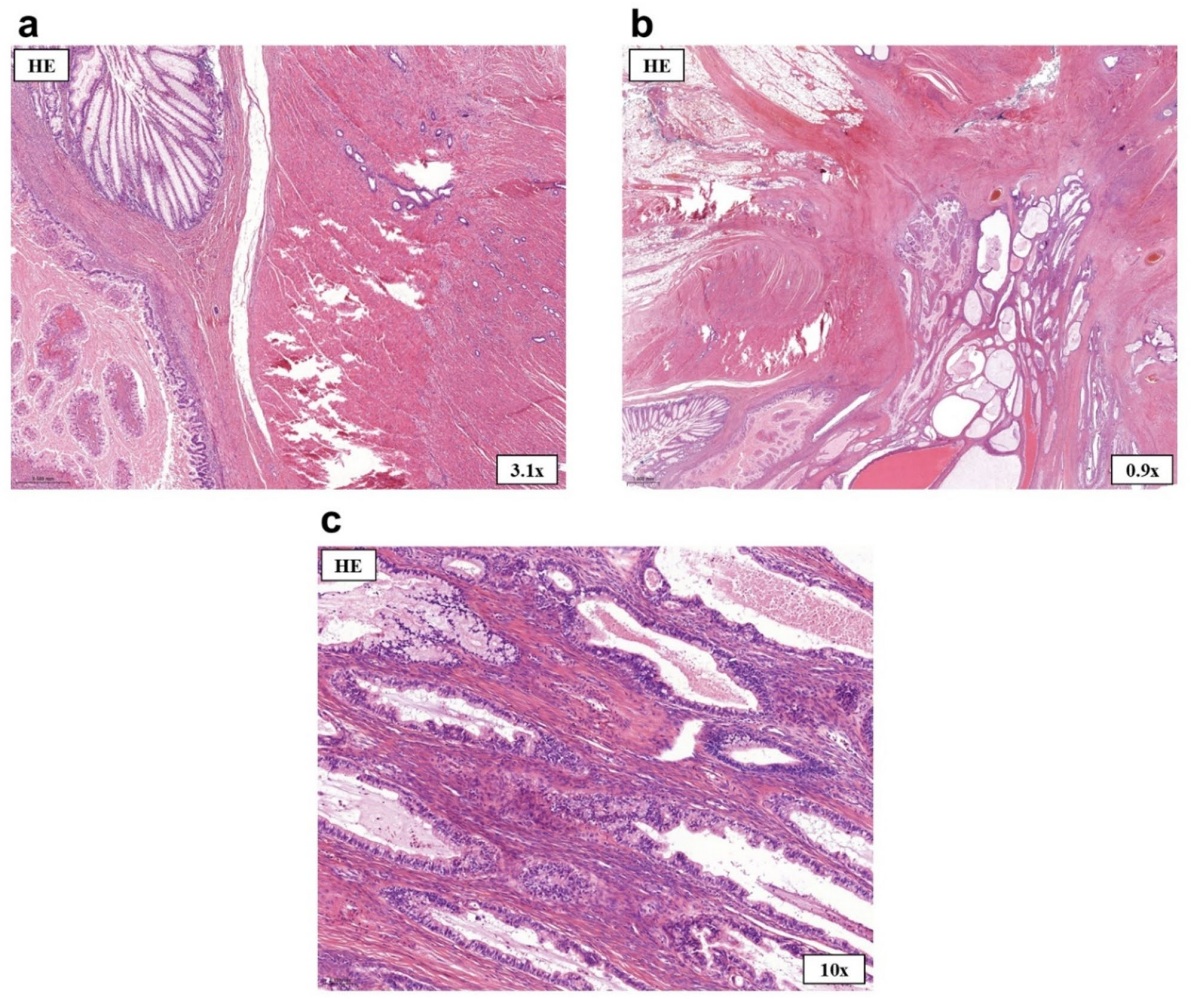
Histopathological examination of the postoperative specimen in Case 2. **(a)** The specimen shows intact colorectal mucosa (upper left corner), endometrioid adenocarcinoma (lower left corner), and endometriosis in the tunica muscularis (upper right area). **(b)** Endometrioid adenocarcinoma with cystically dilated glands. **(c)** Partially borderline and partially low-grade malignant seromucinous endometrioid adenocarcinoma infiltrating the tunica muscularis

**Fig. 7:**
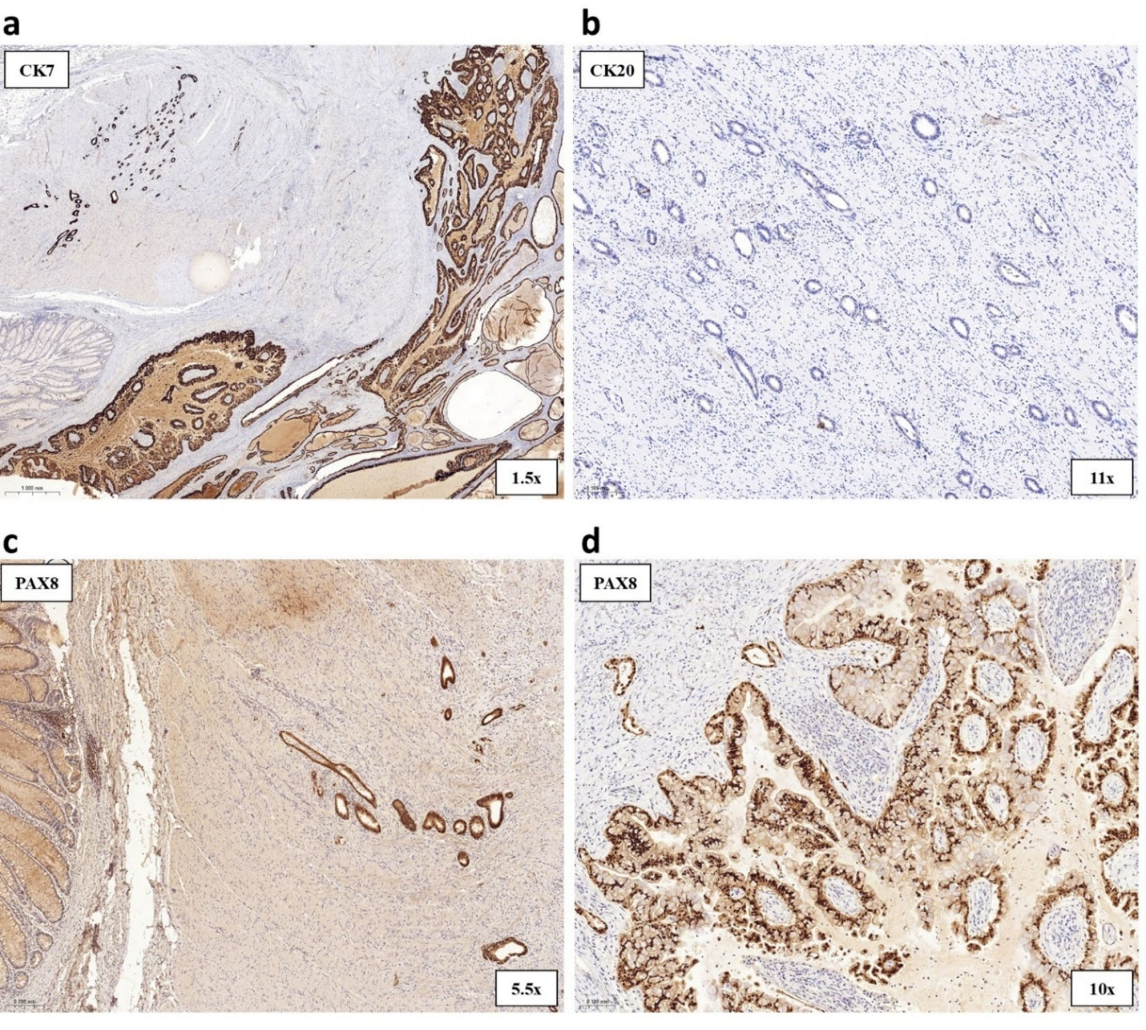
Histopathological examination of the postoperative specimen in Case 2. **(a)** CK7-positivity is observed in both endometriosis within the tunica muscularis (upper left) and infiltrative EAC (lower right). **(b)** The endometrial glands show negative staining for CK20, supporting a noncolorectal origin for the adenocarcinoma, given the concurrent CK7 positivity. **(c–d)** Both endometriosis and endometrioid adenocarcinoma within the tunica muscularis exhibit PAX8 positivity, supporting a Müllerian duct origin

Treatment: The patient underwent laparoscopic sigmoid resection, left oophorectomy, trachelectomy, and omentectomy. Contrary to the initial histopathological diagnosis, final pathology revealed endometriosis infiltrating the entire thickness of the sigmoid colon wall, with a low-grade EAC diffusely spreading within it (Figs. 6a–c and 7a–d). The resection was deemed complete (R_0_), and follow-up imaging showed no evidence of metastasis or recurrence. Under close oncological surveillance, the patient remained asymptomatic and continues to lead an active life.

## Discussion and conclusions

Endometriosis is a common condition affecting women of reproductive age, estimated to affect up to 10% of this population. It is also a significant contributor to chronic pelvic pain, accounting for approximately 70% of cases in women [7]. The pathogenesis of endometriosis has several proposed theories, with Sampson’s theory of retrograde menstruation and the Müllerian remnant hypothesis being widely accepted [8–10]. While endometriotic lesions typically involve internal genital organs, they can extend to various pelvic sites and, less frequently, extrapelvic locations. Among the more unusual sites, involvement of the sciatic nerve, the navel, and the pancreas has been reported [11]. Infiltration of the peritoneal surface of the GI tract is relatively common, with the rectosigmoid colon being the most commonly affected segment. Deeply infiltrating disease (involving the entire bowel wall) is less common but associated with more severe clinical outcomes [6].

GI endometriosis presents with diverse and often nonspecific symptoms, including chronic pelvic or lower abdominal pain, metrorrhagia, menstrual irregularities, and infertility. Physical examination may reveal a palpable pelvic mass [12]. Symptoms vary depending on the lesion’s location, size, and infiltration depth in the GI tract. Common symptoms include dyschezia, dyspareunia, hematochezia, or irritable bowel syndrome-like symptoms. In severe cases, it can cause an acute abdomen due to intussusception, perforation, or bowel obstruction [13–15].

Malignant transformation of GI endometriosis, though extremely rare, represents a clinically significant complication. The most common histological subtype is the EAC, with endometrial stromal sarcomas, particularly in the rectosigmoid region, being exceedingly uncommon [16]. The first documented case of colonic EAC was described by Sampson in 1925 [17]. A review of English-language literature found fewer than 20 reported cases of nonmetastatic colonic EAC (Table 2). These neoplasms pose substantial diagnostic challenges due to their macroscopic resemblance to primary CRC [18]. Most lesions involve the serosa and subserosa, extending variably into deeper layers like the muscularis propria, submucosa, or even the mucosa. Transmural tumors often manifest as a characteristic dumbbell-shaped configuration or as neoplastic polyps. Diagnostic endoscopy may be limited if the mucosa is intact despite submucosal involvement. In such cases, advanced biopsy techniques, such as bite-on-bite or buttonhole sampling, may be necessary [19]. However, lesions with transmural involvement are more accessible for histological sampling.

**Table 2:** A representative collection of English-language publications reporting nonmetastatic colonic EAC cases

Reference	Age of patient	Sexual history	Initial complains	Previous abdominal history	Colonoscopic findings	Radiologic findings	Laboratory findings	Immunhistochemical findings	Surgical treatment
Palla VV et al. (2017) [6]	75	G0	Abdominal pain, GIH	Negative	Bowel intussusception, 28 cm from the anus	**CT**: midline pelvic lesion; two nodal lesions; small cystic lesions in both ovaries **US**: atrophic uterus with thin endometrium	Negative	CK7+, CK20–, Vimentin +	Sigmoidectomy followed by end-to-end anastomy
Jones KD et al. (2002) [28]	52	G0	GIH	TAH and BSO due to rectovaginal endometriosis	A polypoid lesion in the distal sigmoid colon	Did not happen.	n/a	n/a	Stapled anastomosis to the midrectum with a defunctioning loop ileostomy
Smyrniotis et al. (2003) [13]	46	n/a	n/a	Ovarian endometriosis with a chocolate cyst	n/a	n/a	n/a	Vimentin+, CK+, EMA+	Sigmoidectomy with BSO and TAH
Stoklosa et al. (2024) [14]	70	n/a	Abdominal pain	Diverticulosis, partial colectomy, appendectomy, cholecystectomy	Two large purple vascular masses at 10–20 cm from the anal verge, involving 25–50% of the lumen	**CT**: fibroid uterus with two distinct masses	Anemia	CK7+, ER+, PAX8+, CK20-, CDX2-	TAH, BSO, rectosigmoid resection, creation of end colostomy
Kawate et al. (2005) [15]	62	G0	Abdominal pain	TAH and BSO for uterine leiomyoma and pelvic endometriosis	No abnormalities	**CT**: multilocular tumor	Negative	CK7+, CK20–	Sigmoidectomy with lymph node resection
Hoang et al. (2005) [29]	60	G4P3	GIH	TAH and BSO due to extensive endometriosis	3.5 cm sessile polyp on the anterior rectal wall	n/a	n/a	n/a	Sigmoidectomy
Travaglino et al. (2021) [18]	45	n/a	n/a	n/a	n/a	n/a	n/a	**Negative for**: WT1, PAX8, ER, CK20, AFP, CK7, PR **Positive for**: vimentin, EMA, racemase, CDX2, β-catenin, CD10, p16	TAH, BSO and rectosigmoidectomy
Slavin et al. (2000) [16]	49	G0	Abdominal pain, GIH	n/a	n/a	n/a	n/a	n/a	Sigmoidectomy
Slavin et al. (2000) [16]	47	G4P4	Small bowel obstruction	n/a	n/a	n/a	n/a	n/a	Segmental resection of distal ileum
Slavin et al. (2000) [16]	50	G2P2	GIH	n/a	n/a	n/a	n/a	n/a	Segmental resection of small bowel
Amano et al. (1981) [30]	44	Multiparous	Abdominal pain, GIH	Appendectomy and BSO with supravaginal hysterectomy for bilateral endometriosis	Large polypoid tumor on the anterior wall of the sigmoid colon 13 cm from the anus	n/a	n/a	n/a	Sigmoidectomy and end-to-end sigmoid-rectal anastomosis
Ardila-Gatas et al. (2015) [31]	67	n/a	Rectal pain, GIH, weight loss	TAH for endometriosis	No abnormalities	**CT**: 3 × 2.8 cm rectal mass along the anterior right lateral aspect of the rectum. **MRI**: invasion of the superior aspect of the vaginal cuff and a posterior bladder wall mass	n/a	CK7+, ER+, PAX8+, CK20+, CDX2-	Laparoscopic low-anterior resection with a side-to-end colorectal anastomosis, removal of splenic flexure, and diverting loop ileostomy
Duun et al. (1993) [32]	62	G2P2	Climacterial hot flushes	TAH and BSO due to endometriosis	n/a	n/a	n/a		Rectosigmoid resection with colostomy
Lott et al. (1978) [33]	46	G3P3	Abdominal pain, GIH	TAH and BSO	A flat, ulcerating tumor 10 cm from the anal verge, involving a fourth of the circumference of the bowel	n/a	n/a	n/a	Anterior resection of the sigmoid and rectum
Magtibay et al. (2001) [34]	76	n/a	intermittent episodes of diarrhea and GIH	TAH and BSO	Benign hyperplastic polyps	**CT**: 2.5 cm thick cystic mass in the right perirectal region	n/a	n/a	Low anterior resection with colonic J-pouch anal anastomosis and diverting right transverse colostomy
Matías-Garcia et al. (2023) [35]	77	n/a	Abdominal pain, constipation	TAH, BSO, and pelvic lymphadenectomy due to EAC	Stenosing neoplastic mass 20 cm from the anal verge	**CT**: stenosing tumor in the sigmoid colon with locoregional lymphadenopathy	n/a	CK7+, CK20-, ER+	Laparoscopic sigmoidectomy
Reintoft et al. (1974) [36]	36	G1P1	n/a	Right salpingo-oophorectomy and left salpingostomy	Normal mucosa with a bending at the recto-sigmoid junation	**Barium enema**: constriction of several centimetres in length	n/a		Laparoscopic sigmoidectomy

Abbreviations: n/a, not available; GIH, Gastrointestinal hemorrhage; TAH, total abdominal hysterectomy; BSO, bilateral salpingo-oophorectomy; EAC, endometrioid adenocarcinoma

Distinguishing between EAC and CRC is crucial, particularly given their divergent origins: EAC typically invades from the serosal surface inward, while CRC progresses from the mucosa outward [16]. While radiological, endoscopic, and laparoscopic modalities are commonly employed in diagnostic workup, imaging findings are often nonspecific, and even benign endometriotic lesions can be difficult to differentiate from malignant disease. Thus, histopathological examination remains the gold standard for diagnosis. Immunohistochemical staining provides valuable information to distinguish these entities. CRCs are typically CK7-negative and CK20-positive, while EACs are often CK7-positive and CK20-negative (80–100% of cases) [20]. Additional markers like CD10, estrogen, and progesterone receptors can support an endometriotic origin. β-catenin and CD10 positivity can also aid in identifying morular metaplasia [21]. Aberrant cytoplasmic and nuclear accumulation of β-catenin often reflects underlying *CTNNB1* mutations, commonly observed in endometrioid neoplasms. These malignancies typically have a low Ki-67 proliferation index, distinguishing them from other solid tumors. CDX2 expression, while common in GI neoplasms, can also be focally or diffusely positive in gynecological malignancies, potentially leading to diagnostic errors if interpreted alone, particularly in tumors of uncertain primary origin. Given these challenges, a comprehensive immunohistochemical panel, ideally incorporating at least two or more lineage-specific markers and markers associated with metaplasia, is recommended for accurate diagnosis.

Therapeutic options for deeply infiltrating colon endometriosis encompass a broad spectrum. According to Vercellini et al., the evidence supporting the efficacy of hormonal therapy for symptomatic bowel endometriosis is of limited quality, with most available studies being noncomparative and involving heterogeneous treatment regimens and durations [22]. Currently, there are no universally accepted treatment guidelines, and management is largely individualized. Surgical resection is the most common treatment modality, with indications depending on lesion location, infiltration depth, and the degree of luminal obstruction. Surgery is typically the treatment of choice for subacute obstruction.

The completeness of surgical resection is the most critical determinant of recurrence risk. Following R_0_ resection, recurrence ranges between 5 and 15%, whereas incomplete resections (R_1_ or R_2_) may be associated with up to 30%, further influenced by the patient’s hormonal milieu. Hormonal therapy has been shown to reduce the risk of recurrence [23, 24].

In terms of survival, deeply infiltrating endometriosis is a benign condition and, in the absence of malignant transformation, the prognosis is excellent, with near 100% survival [25]. The long-term prognosis in such cases is favorable; however, all patients require ongoing follow-up due to fertility-related issues and intra-abdominal adhesions leading to chronic pelvic pain over time [26]. If transformation to EAC occurs, as seen in Case 2, survival depends on the tumor stage, grade, and the completeness of surgical resection. In early-stage disease (localized, without lymph node involvement), the 5-year survival rate may exceed 90%. In more advanced stages, survival is determined by oncologic factors such as FIGO stage, mitotic index, and the presence of lymphovascular invasion [22].

The cases presented in this work underline the importance of a thorough and meticulous patient workup, as the initial diagnoses can be misleading. In Case 1, the patient was initially suspected to have appendicitis, but further examination revealed the underlying cause of symptoms. Surgery was performed in a semiurgent setting due to critical luminal narrowing, and the intervention was successful due to the relatively well-defined location. The procedure was supported by relevant literature and was considered curative, as there was no evidence of distant metastasis or local invasion [27]. In Case 2, the patient’s history of previous abdominal procedures suggests that scar tissue may have facilitated secondary implantation, supporting the theory of adhesion-mediated spread as a potential etiologic factor. However, the erroneous primary histological diagnosis of serous ovarian carcinoma highlights the importance of careful evaluation and close communication among medical subspecialties, particularly when dealing with rare manifestations of this common disease.

To conclude, these cases highlight the complex pathogenesis and diverse clinical manifestations of endometriosis, reaffirming the need for a thorough differential diagnosis and targeted histopathological evaluation to ensure accurate diagnosis and optimal treatment planning.

### Learning points

1. Always include endometriosis in the differential diagnosis of colorectal masses, especially in women of reproductive age with a history of gynecological symptoms or conditions.
2. Persistent gastrointestinal symptoms in women, as abdominal pain, altered bowel habits, or gastrointestinal bleeding, especially with cyclical nature, warrant thorough evaulation including radiological and endoscopic examination with histopathology.
3. Endoscopic appearance is often non-diagnostic, making successful biopsy essential; however, submucosal lesions often have normal-appearing surface, specialized sampling techniques may be required in such cases.
4. Malignant transformation of gastrointestinal endometriosis is rare but clinically relevant, necessitating precise immunhistochemical evaluation (e.g. CK7+, CK20–, ER+, PR+) to distinguish endometrioid adenocarcinoma from primary colorectal cancer.
5. Surgery should be considered early when obstruction or suspected malignancy is present.

## Data Availability

No datasets were generated or analysed during the current study.

## Abbreviations

CRC: Colorectal carcinoma
CT: Computer tomography
EAC: Endometrioid adenocarcinoma
FOBT: Fecal occult blood test
GI: Gastrointestinal
